# The Operationalization of Gender in Biomedical Research: A Multidimensional Imperative

**DOI:** 10.1007/s10508-025-03230-2

**Published:** 2025-09-08

**Authors:** Irene Göttgens, Aranka Ballering

**Affiliations:** 1 Radboud University Medical Center, Gender Research Unit, Department of Primary and Community Care, Geert Grooteplein Noord 21, 6525 EZ Nijmegen, The Netherlands; 2 University of Groningen, University Medical Center Groningen, Department of Psychiatry, Hanzeplein 1, 9700 RB Groningen, The Netherlands; 3 Ghent University, Faculty of Political and Social Sciences, Department of Sociology, Sint Pietersnieuwstraat 41, 9000 Ghent, Belgium

## Introduction

In the shifting political climate, research that explores variations in sex and gender is increasingly under threat. In the USA, recent federal mandates promote, or rather demand, a fixed and binary definition of sex. This denies lived realities of gender diverse individuals and undermining support for research on sex and gender as determinants of health (Kozlov & Ryan, 2025; The White House, 2025). These mandates reassert a form of biological essentialism and reductionism that has long shaped biomedical research, despite the growing body of evidence that sex and gender, separately and interactively, shape health trajectories and health outcomes.

In response to the call for commentaries, “How many genders are there?”, we expand the focus to what we consider an empirically and methodologically relevant inquiry: “How many gender dimensions are there?” We strongly call for biomedical research that includes gender as a plural, multidimensional, and social construct shaped by context and culture. Biomedical sciences and social sciences often diverge in how explicitly they articulate and deploy gender-related theoretical models to frame research and interpret findings, if done at all. Reductionist approaches in biomedical research have historically struggled to account for lived experiences and social context, not due to their irrelevance, but because such dimensions are difficult to capture within narrowly defined, quantitative frameworks. These approaches often assume that data derived from controlled observation are inherently more valid than insights grounded in conceptual or interpretive frameworks that reflect social, cultural, or experiential realities. This paradigm is based on the notion that scientific observations can be made independently of theory or prior assumptions, a position increasingly challenged in contemporary science (Greenhalgh et al., 2014; Hammarström & Hensing, 2018; Merone et al., 2022a, 2022b).

Although biomedical studies may use “sex” and/or “gender” as a key variables, they primarily do this without engaging with gender theory about what those categories and concomitant constructions mean for (bio)medicine and social structures. This leads to simplistic and deterministic interpretations of findings through categorical thinking, such as men versus women or equating gender with “women” or “trans gender individuals” (Hammarström & Hensing, 2018). Without methodological rigor and a theoretically grounded approach to gender measurement, biomedical research risks inadvertently reinforcing binary understandings of sex and gender, the exact narratives currently being politically mandated and, in doing so, may constrain clinical relevance and accuracy. Drawing on our review of the literature, we find that many dimensions of gender remain insufficiently investigated, leaving key social determinants of health unaccounted for.

## Gender as a Multidimensional Construct

**Fig. 1:**
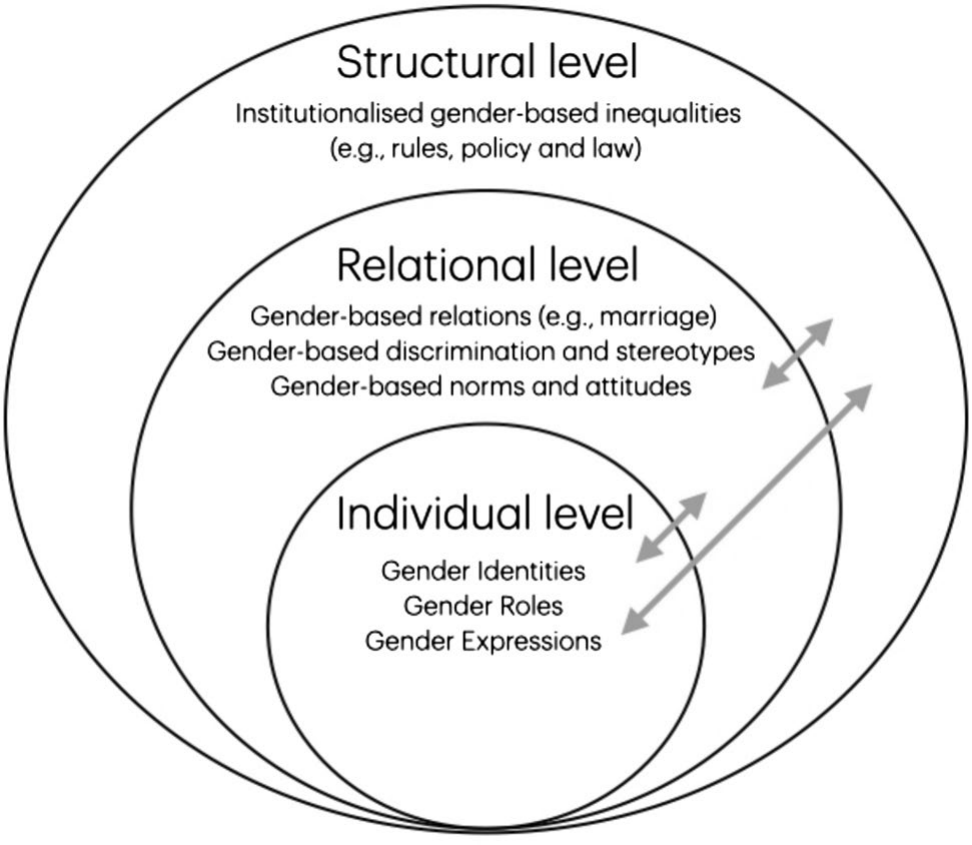
Multidimensionality of gender analyses, adapted from Johnson et al. (2009) and Tadiri et al. (2021)

In “gender performativity” and “doing gender” theory, gender is not considered a singular, stable, identity-based occurrence, but rather a social process that is embedded and embodied in everyday-interactions and societal context (Butler, 1988; West & Zimmerman, 1987). It includes intersecting concepts such as identity, roles, norms, expression, and relational and institutional structures that operate across multiple levels (Fig. 1) (Rotz et al., 2022; Schiebinger et al., 2020). Recognizing and operationalizing these interacting levels in biomedical research is key to identifying gendered pathways that influence health outcomes. Notably, it is a way of resisting dominant, even blatantly mandated, binary framing that simplifies human diversity on a biological and social level (DiMarco et al., 2022).

## Typology of Gender Dimensions

To explore which and how gender dimensions are being measured in biomedical research, we conducted a systematic search in PubMed/MedLine in December 2024 (Online Appendix 1). We retrieved studies that indicated the use of quantitative gender measures among adult participants that were used in (bio)medical studies. Our protocol was preregistered on Open Science Framework (10.17605/OSF.IO/5NGDW). Our final search strategy identified 4651 articles that mentioned gender dimensions and retrieved 63 (1.4%) full-text studies that applied quantitative measures for gender beyond a demographic variable. A total of 60 (1.3%) studies met our inclusion criteria (Online Appendix 2). Table 1 includes the identified dimensions and concepts in biomedical literature and accompanying definitions based on their primary operationalization in the included literature. Depending on their operationalization, gender dimensions are not necessarily limited to a single level.

**Table 1:** Typology of gender dimensions, their level, and definition

Level	Gender dimension	Definition
Individual	Gender identity	Refers to an individual sense-of-self as woman, man, non-binary, queer or by using another term. This does not need to align with one’s sex assigned at birth
	Gender expression	Refers to the external presentation of an individual's gender through behaviors, clothing, hairstyle, voice, and other forms of personal expression
	Gender role attitudes	Refers to individuals' beliefs and perceptions about the appropriate roles, behaviors, and responsibilities for men and women, shaped by societal norms and cultural expectations
	Gender role conflict	Refers to the distress or discomfort experienced when individuals face difficulties in meeting societal expectations associated with their gender roles. This conflict arises when individuals feel that they cannot adhere to traditional gender norms or when they are unable to fulfill the roles expected of their gender identity
Relational	Gender relations	Refers to the ways in which gender and its dimensions influence the interactions, power dynamics, and social relationships between individuals and groups
	Gender norms	Refers to the societal expectations and cultural standards that define what behaviors, traits, and roles are considered appropriate for individuals based on their gender. These norms shape how society expects people to express their masculinity and femininity
	Gender roles	Refers to the social and cultural expectations that dictate the behaviors, responsibilities, and attributes deemed appropriate for individuals based on their gender identity
	Gender-based discrimination	Refers to the unequal or unfair treatment of individuals based on their gender, resulting in limited access to opportunities, resources, or rights. It often manifests through biases, stereotypes, or systemic practices that reinforce gender inequalities
Structural	Institutional/structural gender	Refers to macro-level gender structures that distribute power, resources and opportunities based on individuals’ gender. These structures can be informal or formalized in legislation, guidelines or protocols
	Intersectional gendered experiences	Refers to how gender as a social identity synergistically interacts with other social identities, including but not limited to religion, ethnicity, sexual orientation and socio-economic status, resulting in experiences in which these social identities are intrinsically linked, potentially resulting in a disadvantaged position

Definitions based on Ballering et al. (2023), Johnson et al. (2009), and Tadiri et al. (2021)

## Approaches to Develop Gender Measures

We classified the development of the retrieved gender measures into three main methods (Table 2), with most measures developed via a primary approach. These measures were developed and, in some cases, validated based on pre-defined hypotheses or literature-driven processes. On the one hand, the repeated use of primary approaches supports standardization and comparability across studies by reproducing commonly used gender dimensions and items. This can facilitate cross-study analysis and formation of an evidence base. On the other hand, it also risks reinforcing a self-referential cycle in which the same gender variables are reproduced without critical reflection on their theoretical origins, relevance, or limitations. Over time, this repetition dilutes the conceptual clarity and explanatory strength of gender constructs, reducing them to fixed variables rather than dynamic, context-dependent dimensions. Without explicit theorization, the underlying assumptions embedded in these measures become increasingly opaque, undermining their capacity to meaningfully inform biomedical research and clinical practice.

**Table 2:** Overview of different methods to develop a quantitative gender measure in (bio)medical research

Method	Definition	Example	Frequency of use, *N* (%)
Primary	A gender measure is newly developed and (in some cases) validated via pre-defined hypotheses or a literature-driven approach	Stanford Gender-Related Variables for Health Research (GVHR) (Nielsen et al., 2021)	29 (48.3)
Secondary	A gender measure is developed via a data-driven or theory-driven approach, by using previously collected data. These measures usually describe the adherence of individuals to traditionally masculine and traditionally feminine characteristics	Gender indices, including the Labor Force Gender Index (Smith & Koehoorn, 2016) and Lifelines Gender Index (Ballering et al., 2020)	19 (31.7)
Tertiary	A gender measure is developed by adapting an existing gender measure, for example via a cross-cultural validation	Dutch version of GVHR (Mommersteeg et al., 2025)	12 (20.0)

Irrespective of the type of development, most gender measures rely on dichotomies: a male gender identity or masculinity, in terms of gender roles, stereotypes or expression opposes a female gender identity or femininity as a unidimensional measure. Some of these measures may be based on the “gender diagnosticity approach,” which compares the presence of gendered, i.e., masculine or feminine, traits in individuals to their distribution in male and female populations (Lippa & Connelly, 1990). Unlike many unidimensional gender measures, the Bem Sex Role Inventory (BSRI) conceptualized masculinity and femininity as separate dimensions. Although based on stereotypical traits, it allowed individuals to score high or low on each dimension independently, paving the way for more nuanced understandings of gender expression, such as androgyny (Göttgens et al., 2022; Starr & Zurbriggen, 2017). Furthermore, what seems to be an inherent caveat to most, if not all, gender measures is the lack of dynamicity attributed to gender. The embodiment of gender is a constant (re)negotiation between society’s and individuals’ expectations, making gender a dynamic process embedded in everyday life. Although especially secondary methods are versatile, adaptive, and open to including study-specific societal contexts (Ballering et al., 2024), these measures also cannot fully incorporate the notion of doing gender (West & Zimmerman, 1987).

## Which Gender Dimensions and Concepts Are Measured and Which Are Not?

Based on our review, 98.7% of the screened studies did not measure gender beyond demographical gender identities. The 1.3% that moved beyond mere demographics mainly focused on individual and relational levels through dimensions such as gender identity and gender roles. Gender identity is typically operationalized through self-reported categories, such as man, woman, transgender, non-binary, agender, or gender queer. Gender roles are most frequently quantified via occupational status, caregiving responsibilities, household labor, and instruments such as the BSRI, indicating a wide variety of operationalizations. Sex assigned at birth is often included as a demographic variable in addition to gender identity as an inclusive method for gender diverse populations (two-step method) and as a proxy for biological sex characteristics (Lombardi & Banik, 2016).

Concepts such as gender norms, gender expression, and gender role attitudes are measured less frequently in biomedical studies, but do appear in a subset of included studies. These are typically assessed through items relating to physical and behavioral expressions, and alignment with cultural ideas regarding masculinities and femininities. However, some dimensions remain significantly underused. For example, gender relations are often discussed conceptually and indicated to be important, but rarely integrated as a variable in biomedical literature. Institutionalized or structural gender is rarely included despite being commonly acknowledged as affecting access to (health-related) resources (Acker, 1990; Dore et al., 2024). Similarly, gender-based discrimination, gender role conflict, and intersectional gendered experiences are infrequently included despite their known relevance to health disparities (Homan, 2019; Krieger, 2020). Arguably, intersectional health analyses are neglected due to assumed challenges in measuring participants’ multiple social identities simultaneously (Ballering et al., 2024; Bauer et al., 2021; Turan et al., 2019).

These omissions limit our understanding of how gender operates both as a structural social system and as an embodied determinant of health, shaped by interrelated and heterogeneous dimensions across social and individual levels (Krieger, 2024). Several studies have shown that gender roles and identities, especially individuals’ orientation toward culturally defined masculine and feminine traits, explain more variance in health outcomes than binary classifications based on birth-assigned sex (Ballering et al., 2020, 2022; Göttgens et al., 2022; Pelletier et al., 2015). Moreover, interactions between sex and gender indices show that adherence to masculine or feminine characteristics differently associates with health outcomes for female and male participants. This exemplifies that without measuring distinct gender dimensions, we risk attributing outcomes to biology alone, overlooking the intersecting influences of gendered social norms, identities, and roles.

## Underlying Theories of Gender in Biomedical Research

In theory, biomedical research is increasingly recognizing gender as a sociocultural, plural, and multidimensional construct. Yet, in practice, this recognition remained limited to only 1.3% of identified studies. Most studies continue to define gender narrowly, as a static binary identity, reinforced by a full conflation of gender with sex. Gender is often treated as a psychosocial derivative of biological sex characteristics, rather than a distinct, socially embedded determinant of health. It has been warned that this conflation can introduce misclassification bias, distort health risk estimations, and obscure pathways via which gender shapes health (Ballering et al., 2024; Bauer, 2023; Merone et al., 2022a, 2022b; van den Hurk et al., 2022).

A lack of clear definitions and theoretical positioning severely limits the validity, consistency, and reproducibility of gender-informed biomedical research. The absence of conceptual frameworks in (bio)medicine forces many studies to rely on proxy variables, such as employment or marital roles, without specifically articulating these variables’ relationship to gendered social structures and their health outcome of interest. To advance meaningful gender analysis in biomedical research, precision and multidimensional operationalizations, grounded in theory, are essential to capture the complexity of gendered health disparities and identify modifiable risk factors (Göttgens & Oertelt-Prigione, 2022).

## Rethinking Gender Dimensions for Biomedical Research

Returning to our central question, “How many gender dimensions are there?”; we argue there is at least more than one. Gender dimensions can serve as an analytic lens for examining gendered pathways to health within a multilayered social system (Fig. 1) (Homan, 2019). The relevance of respective dimensions for biomedical research depends on the context, research question, study population, and theoretical orientations. Several reviews suggest that at least five recurring, overarching gender dimensions are slowly finding their way into health research: (1) gender identity; (2) gender expression; (3) gender roles; (4) gender norms/attitudes; (5) institutionalized/structural gender (Ballering et al., 2024; Hartig et al., 2024; Horstmann et al., 2022; van den Hurk et al., 2022). Yet, other dimensions, such as gender relations and intersectional gender experiences are scarcely integrated in biomedical research.

Comprehensive gender analyses do not imply more gender dimensions automatically improve the quality of the research results. Instead, the key is selecting dimensions and survey items that accurately capture the gendered pathways that are hypothesized to affect the health outcome under study. This requires developing *and* integrating robust theoretical models that aim to explain sexed/gendered pathways in health and disease and go beyond binary or demographic proxies (e.g., “female” or “marital status”) (Hammarström & Hensing, 2018). It demands clarity about the purpose of each variable. The central mission for gender analyses in biomedical research is not to count or fix the number of genders (or sexes), but to sharpen how we select, conceptualize and operationalize the dimensions hereof, which enables more clinically relevant, precise and accountable research results.

## (re)Claiming Gender as a Multidimensional Biomedical Variable

The current backlash against integrating sex, gender, or any variety herein in science is not new. Historically, gender’s influence on health outcomes has been overlooked, disregarded, and ignored (Becher & Oertelt-Prigione, 2022; Witt et al., 2024). Today’s political efforts to “restore biological truth” are the latest attempts to dismiss the growing body of evidence showing that gender as a social system profoundly shapes health outcomes, while simultaneously promoting the constraints of biological essentialism through rhetorics of objectivity.

This tension lies at the heart of our work in gender medicine: the use of categories in research is never just descriptive. It is inherently political, shaped by prescriptive, normative practices, and institutional contexts. It requires reflexivity and positionality to recognize that all scientific paradigms in biomedical and health research are embedded in historical, social, and political frameworks. Acknowledging these frameworks does not undermine science, rather it strengthens science by explicating the assumptions that prioritize and legitimize research agendas, and which ultimately determine what knowledge is valued, funded and (re)produced.

How we conceptualize and operationalize gender and its dimensions in research is not a neutral decision either; it shapes what questions can be asked, which (causal) mechanisms are revealed, and whose realities are made visible. In this moment, (re)claiming gender as a multidimensional variable is not only imperative to advancing biomedical knowledge, but also a commitment to epistemic accountability and a defense of scientific integrity. In doing so, we resist the narrowing of inquiry and reaffirm that rigorous, inclusive science thrives not in simplicity, but in its capacity to reflect the full complexity of human experiences.

## Supplementary Information

Below is the link to the electronic supplementary material.

Supplementary file1 (DOCX 48 KB)

## Data Availability

The data on which this commentary is based are made openly available in the supplementary files.
