# Supplementary material for: The Operationalization of Gender in Biomedical Research: A Multidimensional Imperative
Source: Arch Sex Behav. 2025 Sep 8;54(10):3827–32. doi: 10.1007/s10508-025-03230-2 (PMC12753549; doi:10.1007/s10508-025-03230-2)
Supplement: Supplementary file 1 — Supplementary file1 (DOCX 48 KB) [file 10508_2025_3230_MOESM1_ESM.docx]

**Appendix 1**

| **Search strategy** | **Results** |
| --- | --- |
| "gender score"[ti:~3] OR "gender scores"[ti:~3] OR "gender criteria"[ti:~3] OR "gender strategy"[ti:~3] OR “gender strategies”[ti:~3] OR "gender analysis"[ti:~3] OR “gender analyses”[ti:~3] OR "gender scale"[ti:~3] OR “gender scales”[ti:~3] OR "gender integration"[ti:~3] OR “gender integrate”[ti:~3] OR "gender methodology"[ti:~3] OR "gender methods"[ti:~3] OR “gender methodologies”[ti:~3] OR "gender measure"[ti:~3] OR “gender measures”[ti:~3] OR "gender incorporation"[ti:~3] OR “gender incorporate”[ti:~3] OR "gender variables"[ti:~3] OR “gender variable”[ti:~3] OR "gender framework"[ti:~3] OR "gender frame"[ti:~3] OR "gender guideline"[ti:~3] OR "gender guidelines"[ti:~3] OR "gender inclusion"[ti:~3] OR “gender inclusive”[ti:~3] OR "gender approach"[ti:~3] OR “gender approaches”[ti:~3] OR "gender factor"[ti:~3] OR "gender factors"[ti:~3] OR "gender index"[ti:~3] OR "gender indices"[ti:~3] OR "gender dimension"[ti:~3] OR "gender dimensions"[ti:~3] OR "gender lens"[ti:~3] OR “gender perspective”[ti:~3] OR "gender inventory"[ti:~3] OR "gender instrument"[ti:~3] OR “gender instruments”[ti:~3] OR "gender conceptualisation"[ti:~3] OR "gender conceptualization"[ti:~3] OR "gender composition"[ti:~3] OR “gender composite”[ti:~3] OR "gender assessment"[ti:~3] OR "gender technique"[ti:~3] OR "gender survey"[ti:~3] OR "gender questionnaire"[ti:~3] OR "gender associate"[ti:~3] OR “gender account”[ti:~3] OR “gender accounting”[ti:~3] OR “gender considering”[ti:~3] OR “gender consideration”[ti:~3] OR “gender operationalization”[ti:~3] OR “gender operationalisation”[ti:~3]  NOT  "Review"[Publication Type] OR "Systematic Review"[Publication Type] OR "Meta-Analysis"[Publication Type] OR Editorial[pt]  FILTER  Published between 2004 – 2024 | 3923 |

**Appendix 2**

| **Item type** | **Authors** | **Title** | **Which Gender Dimension(s) are addressed** | **Definition of Gender Dimension(s)** | **Primary, secondary or tertiary approach** |
| --- | --- | --- | --- | --- | --- |
|  | Guss CE,Eiduson R,Khan A,Dumont O,Forman SF,Gordon AR | "it'd be great to have the options there": A mixed-methods study of gender identity questions on clinic forms in a primary care setting | Gender identity, gender expression | Gender identity: Self-reported identity, including cisgender, transgender, nonbinary, and genderqueer options. Gender expression: How individuals present their gender through pronouns, names, and social interactions. | Primary approach (hypothesis-driven/literature-driven), implementing the Two-Step Gender Identity Questioning Method recommended for medical settings. |
| **Journal Article** | Pelletier R,Ditto B,Pilote L | A composite measure of gender and its association with risk factors in patients with premature acute coronary syndrome | Gender roles, Gender identity, Gender relations, Institutionalized gender | Gender roles: Behavioral norms applied to men and women in society, influencing daily actions, expectations, and experiences. Gender identity: How individuals see themselves as male, female, or a third gender, affecting behaviors and emotions. Gender relations: Interactions with others based on gender, including how people are treated in social contexts. Institutionalized gender: Distribution of power between genders in social, educational, and political institutions, shaping norms and opportunities. | Secondary approach (data-driven), using principal component analysis (PCA) and logistic regression to construct a gender index. |
| **Journal Article** | Mollborn S,Lawrence EM,Hummer RA | A gender framework for understanding health lifestyles | The study conceptualizes gender as a dynamic multilevel system and incorporates structural sexism as a pathway through which gender shapes health lifestyles​ | The study does not provide explicit definitions for gender roles, gender identity, gender relations, or institutionalized gender but discusses gender as a structural and dynamic factor that interacts with socioeconomic status and life course processes​ | Secondary approach (data-driven), using latent class analysis (LCA) to classify health lifestyles based on gendered patterns​Mollborn et al. 2020 - … |
| **Journal Article** | Corley A,Glass N,Remy MM,Perrin N | A latent class analysis of gender attitudes and their associations with intimate partner violence and mental health in the Democratic Republic of Congo | Gender attitudes towards gender equality and intimate partner violence (IPV), classified into three latent classes | Gender attitudes are conceptualized as views regarding the roles men and women should play in society. These attitudes are shaped by socialization and cultural context and can influence behaviors related to IPV and mental health​ | Secondary approach (data-driven), using latent class analysis (LCA) to identify patterns in gender attitudes​ |
| **Journal Article** | Göttgens I,Darweesh SKL,Bloem BR,Oertelt-Prigione S | A multidimensional gender analysis of health technology self-efficacy among people with Parkinson's disease | Gender Identity, Gender Roles, Gender Relations | Gender Identity: Defined as an individual's self-identification (woman, man, non-binary, or other). Gender Roles: Assessed via gender role orientation, including masculine, feminine, androgynous, or undifferentiated roles. These roles are culturally defined and measured using the Bem Sex Role Inventory (BSRI). Gender Relations: Refers to the ways in which gender influences the distribution of power, responsibilities, and resources within households and society. It was measured through household labor division and relative income compared to a partner. | Primary approach: Hypothesis-driven/literature-driven approach (re-using gender variables from existing literature and measures such as the Bem Sex Role Inventory |
| **Journal Article** | Cipriani E,Samson-Daoust E,Giguère CE,Kerr P,Consortium,Lepage C,Juster RP | A step-by-step and data-driven guide to index gender in psychiatry | Gender identity, gender relations, gender roles, institutionalized gender | Gender identity: How individuals self-identify, behave, express gender, and are perceived by others. Gender relations: Social interactions and perceptions of gendered identity. Gender roles: Social expectations and norms associated with a given gender. Institutionalized gender: Distribution of power, resources, and opportunities based on gender. | Secondary approach (data-driven), using exploratory factor analysis (EFA) and confirmatory factor analysis (CFA) to create a composite gender score. |
| **Journal Article** | Sedlander E,Dahal M,Bingenheimer JB,Puri MC,Rimal RN,Granovsky R,Diamond-Smith NG | Adapting and validating the G-NORM (gender norms scale) in Nepal: An examination of how gender norms are associated with agency and reproductive health outcomes | Gender norms, specifically descriptive and injunctive gender norms | Descriptive norms: Individuals' beliefs about what people in their community are doing (e.g., whether most parents in the community pay a dowry for their daughter's marriage). Injunctive norms: Individuals' beliefs about what people in their community think they should do (e.g., whether they should pay a dowry when their daughter is married). | Tertiary approach (adaptation), as the G-NORM scale was originally developed in India and then adapted and validated for Nepal. |
| **Journal Article** | Green L,Rimes KA,Rahman Q | Beliefs About Others' Perceptions-Gender Typicality: Scale development and relationships to gender nonconformity, sexual orientation, and well-being | Gender typicality, gender nonconformity, and beliefs about others’ perceptions of gender | Gender typicality: How individuals believe others perceive their gender expression in terms of appearance, behavior, and interests. Gender nonconformity: The degree to which an individual’s behaviors, interests, and self-presentation deviate from societal gender norms. | Primary approach (hypothesis-driven/literature-driven), with scale items based on prior gender nonconformity research and gender role measurement. |
| **Journal Article** | Fraser G,Bulbulia J,Greaves LM,Wilson MS,Sibley CG | Coding responses to an open-ended gender measure in a New Zealand national sample | Gender identity and gender diversity | Gender identity: How individuals self-identify their gender, including non-binary, transgender, and culturally specific gender identities. Gender diversity: Variations in gender identity beyond the binary classification of male and female, captured through an open-ended measure. | Secondary approach (data-driven), as gender responses were categorized based on emergent coding from qualitative data. |
| **Journal Article** | Lacasse A,Pagé MG,Choinière M,Dorais M,Vissandjée B,Nguefack HLN,Katz J,Samb OM,Vanasse A,TORSADE Cohort Working Group | Conducting gender-based analysis of existing databases when self-reported gender data are unavailable: the GENDER Index in a working population | Gender roles, gender relations, institutionalized gender | Gender roles: Behavioral norms applied to men and women, influencing occupation, childcare responsibilities, and work-related stress. Gender relations: How individuals interact with and are treated by others based on their gender, reflected in community belonging and social stressors. Institutionalized gender: The distribution of power and resources between men and women, captured through variables like income, education, and job sector. | Secondary approach (data-driven), using propensity scoring from logistic regression models to create a gender index. |
| **Journal Article** | van Well S,Kolk AM,Arrindell WA | Cross-cultural validity of the masculine and feminine gender role stress scales | Gender roles and gender role stress. | Gender role stress is defined as stress resulting from perceived failure to meet cultural expectations associated with one's gender role. The study examines how rigid adherence to gender role norms contributes to stress in men and women. The Masculine Gender Role Stress (MGRS) scale assesses stress related to failing to meet masculine expectations, while the Feminine Gender Role Stress (FGRS) scale measures stress related to feminine role expectations. | Tertiary approach: translation of existing measures (Masculine Gender Role Stress (MGRS) Scale by Eisler & Skidmore, 1987, and Feminine Gender Role Stress (FGRS) Scale by Gillespie & Eisler, 1992) from English into Dutch, with back-translation to ensure equivalence. |
| **Journal Article** | Dandolo L,Hartig C,Telkmann K,Horstmann S,Schwettmann L,Selsam P,Schneider A,Bolte G,On Behalf Of The Inger Study Group | Decision tree analyses to explore the relevance of multiple sex/gender dimensions for the exposure to green spaces: Results from the KORA INGER study | Sex assigned at birth, gender identity, internalized gender roles, externalized gender expressions, discrimination experiences, care activities, intersectionality-related social categories | Sex assigned at birth: Participant’s recorded sex at birth. Gender identity: Self-reported gender identity, including multiple response options. Internalized gender roles: How individuals perceive themselves in terms of masculinity and femininity. Externalized gender expressions: How individuals believe others perceive their gender based on appearance and behavior. Discrimination experiences: Self-reported experiences of discrimination based on various attributes, including gender, ethnicity, and socioeconomic status. Care activities: Household and caregiving responsibilities as measures of gender roles. Intersectionality-related social categories: Socioeconomic indicators such as education level, income, and employment status. | Secondary approach (data-driven), using decision tree analyses (CART and CIT) to explore the relevance of multiple sex/gender covariates for exposure to green spaces |
| **Journal Article** | Fleming PJ,Harris KM,Halpern CT | Description and evaluation of a measurement technique for assessment of performing gender | Gender performance, gender typicality, doing gender | Gender performance: The extent to which an individual's behaviors align with those of their same-gender peers, measured as a continuous probability score. Gender typicality: The degree to which an individual's reported behaviors are consistent with social expectations of their gender. Doing gender: A dynamic, interactional process where individuals enact behaviors that align with dominant gender norms. | Secondary approach (data-driven), using logistic regression and probability scoring to quantify gender performance based on behavioral patterns |
| **Journal Article** | Simbar M,Rahmanian F,Nazarpour S,Ramezankhani A,Eskandari N,Zayeri F | Design and psychometric properties of a questionnaire to assess gender sensitivity of perinatal care services: a sequential exploratory study | Gender sensitivity, gender roles in perinatal care, gender-sensitive health services | Gender sensitivity: The responsiveness of perinatal care services to the specific health needs of men and women based on their gender roles. Gender roles: The influence of societal expectations on the involvement of men and women in perinatal healthcare. | Primary approach (hypothesis-driven/literature-driven), using qualitative content analysis and literature review to develop questionnaire items |
| **Journal Article** | Yang X,Li S,Wu Z,Schimmele CM | Developing scales for measuring gender behaviors in reproductive health in rural China | Gender Roles, Gender Relations | Gender Roles: Defined as the responsibilities, expectations, and behaviors assigned to men and women in the context of family planning and reproductive health. The study particularly focuses on gendered participation in contraceptive use, reproductive decision-making, and caregiving responsibilities. Gender Relations: Defined as the interactions between men and women within the FP/RH system, including decision-making power within households and how gender impacts client-provider interactions in FP/RH services. The study examines whether service providers treat clients differently based on gender and whether FP policies reinforce existing gender inequalities. | Primary approach: Literature-driven approach, as the items were developed through focus groups, in-depth interviews, and prior studies (e.g., Orlofsky’s Sex Role Behavior Scale, McGrath’s Masculine-Feminine Pathology Scale). |
| **Journal Article** | Schwartz-Salazar S,García-Sánchez E,Martínez R,Rodríguez-Bailón R | Development and validation of the Multidimensional Gender Inequality Perception Scale (MuGIPS) | Perceived gender inequality across multiple domains: health, violence, household work and caregiving, public sphere and power (including education, paid job and economics, and political representation) | Perceived gender inequality: Awareness of the existence of differences between women and men in roles, opportunities, and outcomes across life domains (health, violence, caregiving, employment, and public life). Health: Differences in medical attention, research gaps, and misdiagnosis patterns. Violence: Disproportionate exposure of women to harassment, intimate partner violence, and assault. Household work and caregiving: Unequal burden of unpaid caregiving responsibilities on women. Public sphere and power: Gender gaps in education, labor market participation, wages, and political representation. | Primary approach (hypothesis-driven/literature-driven), based on existing gender inequality indices (e.g., Gender Equality Index, Gender Inequality Index) and expert consultation. |
| **Journal Article** | Wandschneider L,Sauzet O,Razum O,Miani C | Development of a gender score in a representative German population sample and its association with diverse social positions | Gendered social practices, relational gender, intersectionality in gender analysis | Gendered social practices: Daily behaviors and societal expectations that reflect gendered norms. Relational gender: Gender as a dynamic and context-specific construct influenced by interactions, power structures, and societal roles. Intersectionality: How gendered practices vary by social categories such as migration status, socioeconomic status, and relationship status. | Secondary approach (data-driven), applying multivariable logistic regression and conditional probabilities to generate a gender score based on gendered social practices. |
| **Journal Article** | Islam A,Anwar Siraji M,Haque M,Salim Chowdhury M | Development of a multidomain gender norm attitude scale for youth in Bangladesh | Gender norms, gender roles, gender-based decision-making | Gender norms: Social expectations and rules that define appropriate behavior for different genders. Gender roles: Expected responsibilities and societal positions assigned to individuals based on gender. Gender-based decision-making: Influence of gender on household, financial, and career-related choices. | Primary approach (hypothesis-driven/literature-driven), based on literature review, qualitative data, and expert consultation. |
| **Journal Article** | Swartout KM,Parrott DJ,Cohn AM,Hagman BT,Gallagher KE | Development of the abbreviated Masculine Gender Role Stress scale | Masculine gender role stress (MGRS) | Masculine Gender Role Stress (MGRS): The psychological strain experienced by men when they perceive themselves as failing to meet traditional male gender norms, particularly in areas such as physical adequacy, emotional expression, subordination to women, intellectual inferiority, and performance failure. | Secondary approach (data-driven), using item response theory (IRT) and classical test theory (CTT) to refine and validate the scale. |
| **Journal Article** | García-Cueto E,Rodríguez-Díaz FJ,Bringas-Molleda C,López-Cepero J,Paíno-Quesada S,Rodríguez-Franco L | Development of the Gender Role Attitudes Scale (GRAS) amongst young Spanish people | Gender role attitudes, sexism, and gender equality perspectives | Gender role attitudes: Beliefs and perceptions about gender roles that influence social behavior and relationships. Sexism: Traditional beliefs that reinforce gender stereotypes and justify gender-based discrimination. Gender equality perspectives: Attitudes that support equal treatment and opportunities for all genders. | Primary approach (hypothesis-driven/literature-driven), based on existing scales and theoretical perspectives on gender equality. |
| **Journal Article** | Lagos D,Compton D | Evaluating the use of a two-step gender identity measure in the 2018 General Social Survey | Gender identity, sex assigned at birth, external gender perception | Gender identity: Self-reported current gender, including options for woman, man, transgender, or a gender not listed Sex assigned at birth: The sex assigned to the respondent at birth, as indicated on their birth certificate (male, female, intersex) External gender perception: Interviewer-coded sex, which can differ from self-reported gender identity | Primary approach (hypothesis-driven/literature-driven), based on prior research supporting the two-step gender identity measure as a valid way to differentiate sex assigned at birth from gender identity |
| **Journal Article** | Wesson PD,Lippman SA,Neilands TB,Ahern J,Kahn K,Pettifor A | Evaluating the validity and reliability of the Gender Equitable Men's Scale using a longitudinal cohort of adolescent girls and young women in South Africa | Gender norms, gender equity, gender-based power dynamics | Gender norms: Social expectations that define appropriate behaviors for men and women, influencing health and risk behaviors. Gender equity: Attitudes toward fairness and equal opportunities for women and men in social, familial, and economic roles. Gender-based power dynamics: The impact of gendered social structures on decision-making, autonomy, and experiences of intimate partner violence (IPV). | Secondary approach (data-driven), using Item Response Theory (IRT) and measurement invariance analysis to validate the longitudinal performance of the scale. |
| **Journal Article** | Fernández J,Quiroga MÁ,Escorial S,Privado J | Explicit and implicit assessment of gender roles | Gender roles, gender stereotypes | Gender roles: Specific activities more frequently performed by men or women in a given society and era, categorized into work and domestic spheres. Gender stereotypes: Beliefs and attitudes about perceived differences between men and women, shaping expectations about appropriate behaviors for each gender. | Primary approach (hypothesis-driven/literature-driven), based on previous theoretical distinctions between explicit and implicit gender role assessments. |
| **Journal Article** | Ghodrati M,Walton DM,MacDermid JC | Exploring the domains of gender as measured by a new gender, pain and Expectations Scale | Gender traits, gender role expectations, pain-related gender norms | Gender traits: Personality characteristics traditionally associated with masculinity or femininity, influencing behavior and self-perception. Gender role expectations: Societal norms dictating how individuals of different genders should behave in response to pain and emotional expression. Pain-related gender norms: Cultural assumptions about differences in pain sensitivity, tolerance, and willingness to report pain based on gender. | Primary approach (hypothesis-driven/literature-driven), integrating items from the Bem Sex Role Inventory (BSRI) and the Gender Role and Expectations of Pain (GREP) Scale to create the new Gender, Pain and Expectations Scale (GPES). |
| **Journal Article** | Zhang C,Blashill AJ,Wester SR,O'Neil JM,Vogel DL,Wei J,Zhang J | Factor structure of the Gender Role Conflict Scale-Short Form in Chinese heterosexual and gay samples | Gender role conflict (GRC) | Gender role conflict (GRC): Psychological stress experienced when men feel they are failing to meet societal expectations of masculinity. GRC occurs when rigid adherence to traditional male norms creates distress in emotional expression, relationships, and career-life balance. | Tertiary approach (adaptation), as the Gender Role Conflict Scale-Short Form (GRCS-SF) was translated from English into Chinese and adapted for use with Chinese men. |
| **Journal Article** | Lusey H,San Sebastian M,Christianson M,Edin KE | Factors associated with gender equality among church-going young men in Kinshasa, Democratic Republic of Congo: a cross-sectional study | Gender equality, gender norms, masculinity beliefs | Gender equality: The belief in equal rights, responsibilities, and opportunities for men and women. Gender norms: Societal expectations regarding appropriate behavior for men and women, particularly regarding domestic roles, sexuality, and masculinity. Masculinity beliefs: Perceptions of male dominance, toughness, and sexual entitlement. | Primary approach (hypothesis-driven/literature-driven), using adapted survey items from the International Men and Gender Equality Survey (IMAGES) and the Gender-Equitable Men (GEM) scale |
| **Journal Article** | Ballering AV,Bonvanie IJ,Olde Hartman TC,Monden R,Rosmalen JGM | Gender and sex independently associate with common somatic symptoms and lifetime prevalence of chronic disease | Gender roles, institutionalized gender | Gender roles: Socially prescribed behaviors and expectations associated with masculinity and femininity, influencing work participation, household responsibilities, and personality traits. Institutionalized gender: Structural and systemic factors influencing opportunities and constraints based on gender, such as job sector, education, and caregiving responsibilities. | Secondary approach (data-driven), using LASSO logistic regression to derive a gender index from existing cohort data. |
| **Journal Article** | Shamasneh B,Nemer M,Abu-Rmeileh NME | Gender awareness in healthcare: Contextualization of an Arabic version of the Nijmegen Gender Awareness in Medicine Scale (N-GAMS) | Gender awareness, gender sensitivity, gender role ideology | Gender awareness: Understanding the socially determined differences between men and women and how they affect access to healthcare and medical treatment. Gender sensitivity: The degree to which healthcare professionals recognize and address gender differences in medical practice. Gender role ideology: Beliefs about gender roles, including attitudes toward gendered interactions with patients and colleagues. | Tertiary approach (adaptation), translating and adapting the Nijmegen Gender Awareness in Medicine Scale (N-GAMS) into Arabic. |
| **Journal Article** | Morais R,Bernardes SF,Verdonk P | Gender awareness in medicine: adaptation and validation of the Nijmegen Gender Awareness in Medicine Scale to the Portuguese population (N-GAMS) | Gender awareness, gender sensitivity, gender-role ideology towards patients, gender-role ideology towards doctors | Gender awareness: The ability to recognize and integrate gender-related differences in medical practice. Gender sensitivity: Awareness and responsiveness to gender differences in healthcare and medical treatment. Gender-role ideology towards patients: Stereotypical beliefs about male and female patients, influencing physician perceptions and treatment decisions. Gender-role ideology towards doctors: Stereotypical beliefs about male and female physicians, influencing workplace dynamics in medicine. | Tertiary approach (adaptation), adapting and validating the Nijmegen Gender Awareness in Medicine Scale (N-GAMS) for the Portuguese population. |
| **Journal Article** | Cummings S,Ramage K,Scime NV,Ahmed SB,Brennand EA | Gender expression is associated with selection of uterine preservation or hysterectomy for pelvic organ prolapse surgery: Novel methodology for sex- and gender-based analysis in gynecologic research | Gender expression | Gender expression: How individuals present their gender through appearance, behavior, or physical attributes, measured through self-reported femininity and masculinity scores. | Primary approach (hypothesis-driven/literature-driven), based on the Magliozzi gender expression tool, which assesses self-rated femininity and masculinity. |
| **Journal Article** | Herdman KJ,Choi N,Fuqua DR,Newman JL | Gender role conflict scale: validation for a sample of gay men and lesbian women | Gender role conflict | Gender role conflict: Psychological distress arising from rigid adherence to traditional gender roles, which can affect cognitive, emotional, and behavioral experiences. | Tertiary approach (adaptation), validating the Gender Role Conflict Scale (GRCS) for use with gay men and lesbian women. |
| **Journal Article** | Haarmans M,McKenzie K,Kidd SA,Bentall RP | Gender role strain, core schemas, and psychotic experiences in ethnically diverse women: A role for sex- and gender-based analysis in psychosis research? | Gender role strain (GRS), femininity ideology, implicit gender stereotypes | Gender Role Strain (GRS): Psychological distress experienced due to perceived discrepancies between self-concept and gender-role expectations. Femininity Ideology: Endorsement of traditional beliefs about femininity, including roles in caretaking, purity, and emotionality. Implicit Gender Stereotypes: Unconscious associations between gender and personality traits or behaviors. | Primary approach (hypothesis-driven/literature-driven), integrating prior research on gender and psychosis. |
| **Journal Article** | Nauman AT,Behlouli H,Alexander N,Kendel F,Drewelies J,Mantantzis K,Berger N,Wagner GG,Gerstorf D,Demuth I,Pilote L,Regitz-Zagrosek V | Gender score development in the Berlin Aging Study II: a retrospective approach | Gender identity, gender roles, gender relations, institutionalized gender | Gender identity: How individuals perceive themselves and experience their gender, measured using chronic stress, perceived stress, personality traits (Big Five: agreeableness, neuroticism, extraversion), and risk-taking behavior. Gender roles: Socially assigned responsibilities and behaviors based on gender, measured through employment status. Gender relations: Social connections and interactions influenced by gender, assessed via loneliness. Institutionalized gender: Structural factors shaping gender experiences, measured through education level and family status. | Secondary approach (data-driven), using principal component analysis (PCA) and logistic regression to develop a gender score from retrospective data. |
| **Journal Article** | Smits RLA,van Dongen LH,Blom MT,Tan HL,van Valkengoed IGM | Gender-related factors and out-of-hospital cardiac arrest incidence in women and men: analysis of a population-based cohort study in the Netherlands | Gender-related factors in employment, economic responsibility, caregiving, and social relations | Employment status: Whether an individual was employed, unemployed, or a pensioner. Economic responsibility (Primary Earner Status): The extent to which an individual contributes to household income (primary earner, equal earner, or not a primary earner). Caregiving burden (Living with Children): Presence of children (<18 years) in the household. Social relations (Marital Status & Living Situation): Whether an individual was married, not married, divorced, or widowed, and whether they lived alone or with others. | Secondary approach (data-driven), using administrative cohort data and Cox proportional hazards models to estimate associations between gender-related factors and OHCA risk. |
| **Journal Article** | Nielsen MW,Stefanick ML,Peragine D,Neilands TB,Ioannidis JPA,Pilote L,Prochaska JJ,Cullen MR,Einstein G,Klinge I,LeBlanc H,Paik HY,Schiebinger L | Gender-related variables for health research | Gender norms, gender-related traits, gender relations | Gender norms: Social expectations shaping behaviors and responsibilities based on gender. Gender-related traits: Personality characteristics and behaviors associated with masculinity and femininity. Gender relations: Social and economic interactions influenced by gendered power structures. | Primary approach (hypothesis-driven/literature-driven), based on systematic review and expert consultation. |
| **Journal Article** | Michaels S,Milesi C,Stern M,Viox MH,Morrison H,Guerino P,Dragon CN,Haffer SC | Improving measures of sexual and gender identity in English and Spanish to identify LGBT older adults in surveys | Gender identity, gender expression, and sexual orientation | Gender identity: Self-reported gender, including male, female, transgender, or a non-binary identity. Gender expression: Presentation of gender through behaviors and appearance. Sexual orientation: Self-identification as heterosexual, lesbian/gay, bisexual, or another sexual identity. | Tertiary approach (adaptation), evaluating and modifying existing survey measures for gender and sexual identity to improve comprehension among older adults. |
| **Journal Article** | Mena E,Stahlmann K,Telkmann K,Bolte G,On Behalf Of The AdvanceGender Study Group | Intersectionality-informed sex/gender-sensitivity in public health monitoring and reporting (PHMR): A case study assessing stratification on an "Intersectional Gender-Score" | Intersectional gender score (IG-Score), gender roles, institutionalized gender | Intersectional Gender-Score (IG-Score): A probability-based measure estimating the likelihood of "being a woman" based on sociocultural, sociodemographic, and socioeconomic factors. Gender roles: Employment type, caregiving roles, and social expectations tied to gender. Institutionalized gender: Structural aspects influencing gender, such as occupational segregation and economic participation. | Secondary approach (data-driven), using Gradient Boosting Models (GBM) and logistic regression to develop an Intersectional Gender-Score (IG-Score). |
| **Journal Article** | Sánchez-Rodríguez Á,Moreno-Bella E,García-Sánchez E | Mapping gender stereotypes: a network analysis approach | Gender stereotypes, gender metastereotypes, in-group stereotypes | Gender stereotypes: Shared societal beliefs about attributes associated with men and women. Gender metastereotypes: Beliefs individuals have about how their gender group is perceived by members of the opposite gender. In-group stereotypes: Stereotypical attributes individuals apply to their own gender group. | Primary approach (hypothesis-driven/literature-driven), using a network analysis method to examine the structure of gender stereotypes based on spontaneous responses. |
| **Journal Article** | Walter JG | Measures of gender role attitudes under revision: The example of the German General Social Survey | Gender role attitudes, gender ideology, division of labor in the family | Gender role attitudes: Beliefs about the roles and responsibilities assigned to men and women in work and family life. Gender ideology: Perspectives on traditional versus egalitarian gender norms in employment and caregiving. Division of labor in the family: Views on the breadwinner model, father’s role in childcare, and shared household responsibilities. | Tertiary approach (adaptation), revising existing gender role attitude measures to align with contemporary gender roles. |
| **Journal Article** | Smith PM,Koehoorn M | Measuring gender when you don't have a gender measure: constructing a gender index using survey data | Gender roles, institutionalized gender | Gender roles: Behavioral norms applied to men and women, reflected in workforce participation, caregiving responsibilities, and occupational segregation. Institutionalized gender: How power and opportunities are distributed based on gender, operationalized through differences in education levels and employment patterns. | Secondary approach (data-driven), constructing a Labour Force Gender Index (LFGI) using survey data. |
| **Journal Article** | Fernández J,Quiroga MA,del Olmo I,Aróztegui J,Martín A | Objective assessment of gender roles: Gender Roles Test (GRT-36) | Gender roles, gender stereotypes | Gender roles: Socially assigned responsibilities and tasks traditionally associated with men and women, categorized into domestic and work spheres. Gender stereotypes: Implicit associations between gender and specific activities, measured through reaction times (RTs) in a decision-making task. | Primary approach (hypothesis-driven/literature-driven), based on gender role theory and social cognition models. |
| **Journal Article** | Baiocco R,Antoniucci C,Basili E,Pistella J,Favini A,Martin C,Pastorelli C | Perceived Similarity to Gender Groups Scale: Validation in a sample of Italian LGB + and heterosexual young adults | Gender typicality, gender similarity | Gender typicality: Perceived similarity to one’s own gender group in behaviors, interests, and social interactions. Gender similarity: Dual-identity concept assessing both own-gender similarity and other-gender similarity as independent constructs. | Primary approach (hypothesis-driven/literature-driven), based on Martin et al. (2017), expanding the Perceived Similarity to Gender Groups Scale (PSGGS) to a young adult population. |
| **Journal Article** | Opekitan AT,Ogunsemi O,Osalusi B,Adeleye O,Ale A | Perception of victims of rape and perception of gender social roles among college students in Southwest Nigeria: validation of a 5-item gender scale | Gender roles, institutionalized gender | Gender roles: Socially assigned responsibilities and expectations associated with men and women, particularly in work, household, and leadership. Institutionalized gender: Structural and societal beliefs about gendered expectations in family and occupational roles. | Tertiary approach (adaptation), modifying the 10-item Gender Role Belief Scale into a 5-item Gender Social Scale for use in Nigerian college students. |
| **Journal Article** | Bolijn R,Kunst AE,Appelman Y,Galenkamp H,Moll van Charante EP,Stronks K,Tan HL,van Valkengoed IG | Prospective analysis of gender-related characteristics in relation to cardiovascular disease | Gender roles, gender relations, institutionalized gender | Gender roles: Expectations regarding division of labor, caregiving, and occupational engagement (e.g., employment type, household work, home repairs). Gender relations: Social interactions and dependency structures influencing health (e.g., emotional support needs). Institutionalized gender: Structural inequalities in the labor market and economic participation (e.g., primary earner status, male- vs. female-dominated occupations). | Secondary approach (data-driven), using cohort survey data to construct gender-related variables and analyze associations with cardiovascular disease (CVD). |
| **Journal Article** | Celebi Cakiroglu O,Harmanci Seren AK | Psychometric properties of the Gender Role Attitudes Scale among Turkish nursing students and factors affecting their attitudes | Gender roles, gender role attitudes | Gender roles: Socially and culturally constructed expectations regarding behaviors and responsibilities assigned to men and women. Gender role attitudes: Beliefs and perspectives on traditional versus egalitarian gender roles in family, work, and social settings. | Tertiary approach (adaptation), adapting the Gender Role Attitudes Scale (GRAS) into Turkish (GRAS-TR). |
| **Journal Article** | Gruber FM,Distlberger E,Scherndl T,Ortner TM,Pletzer B | Psychometric properties of the multifaceted gender-Related Attributes Survey (GERAS) | Gender role identity, gender stereotypes, masculinity, femininity | Gender role identity: An individual’s identification with culturally defined masculinity or femininity. Gender stereotypes: Social expectations about behaviors, traits, and interests associated with men and women. Masculinity and femininity: Conceptualized as separate dimensions encompassing personality traits, cognitive abilities, and interests. | Primary approach (hypothesis-driven/literature-driven), integrating multiple theoretical perspectives to develop a multidimensional measure of gender-related attributes. |
| **Journal Article** | Alipour P,Azizi Z,Raparelli V,Norris CM,Kautzky-Willer A,Kublickiene K,Herrero MT,Emam KE,Vollenweider P,Preisig M,Clair C,Pilote L | Role of sex and gender-related variables in development of metabolic syndrome: A prospective cohort study | Gender identity, gender roles, gender relations, institutionalized gender | Gender identity: Self-reported male or female classification used in sex-stratified analysis. Gender roles: Employment status, caregiving responsibilities, household work, and occupational level. Gender relations: Marital/partner status, social support, and financial dependency. Institutionalized gender: Socioeconomic status indicators including education level and income category. | Secondary approach (data-driven), using logistic regression and multivariable modeling to analyze the impact of gender-related variables on metabolic syndrome (MetS). |
| **Journal Article** | Wylie SA,Corliss HL,Boulanger V,Prokop LA,Austin SB | Socially assigned gender nonconformity: A brief measure for use in surveillance and investigation of health disparities | Socially assigned gender nonconformity, gender expression, gender-related victimization | Socially assigned gender nonconformity: How an individual’s gender expression is perceived by others, independent of their own gender identity. Gender expression: The way people present their gender through appearance and mannerisms, assessed based on social perceptions. Gender-related victimization: Experiences of discrimination, bullying, or violence based on gender nonconformity. | Primary approach (hypothesis-driven/literature-driven), developing a brief self-report measure of socially assigned gender nonconformity for public health research. |
| **Journal Article** | Barth A,Trübner M | Structural stability, quantitative change: A latent class analysis approach towards gender role attitudes in Germany | Gender roles, gender role attitudes, family roles | Gender roles: Social expectations regarding appropriate behaviors and responsibilities for men and women. Gender role attitudes: Beliefs about the division of labor in the family and workforce, particularly concerning working mothers. Family roles: Expectations about mothers' and fathers' responsibilities in childcare and employment. | Secondary approach (data-driven), using latent class analysis (LCA) to cluster gender role attitudes. |
| **Journal Article** | Sedlander E,Bingenheimer JB,Long MW,Swain M,Rimal RN | The G-NORM scale: Development and validation of a theory-based gender norms scale | Gender norms, gender expectations, household power, gendered decision-making | Gender norms: Social expectations regarding acceptable and appropriate behaviors for men and women. Gender expectations: Cultural beliefs about gendered responsibilities, particularly in household labor and caregiving. Household power and decision-making: Gendered authority in financial and mobility decisions within families. Other-oriented norms: The expectation that women prioritize family needs over their own. | Primary approach (hypothesis-driven/literature-driven), integrating social norms theory and gender and power theory to create a theory-based gender norms scale. |
| **Journal Article** | Göttgens I,Darweesh SKL,Bloem BR,Oertelt-Prigione S | The impact of multiple gender dimensions on health-related quality of life in persons with Parkinson's disease: an exploratory study | Gender identity, gender roles, gender relations | Gender identity: A person’s self-reported identity (e.g., man, woman, non-binary), separate from sex assigned at birth. Gender roles: Stereotypical behaviors, roles, and attitudes culturally associated with men and women, measured through gender expression and gender role orientation. Gender relations: How gender influences social interactions, division of household labor, caregiving responsibilities, and interactions in medical settings. | Primary approach (hypothesis-driven/literature-driven), integrating theoretical frameworks and validated scales to measure gender dimensions in PD patients. |
| **Journal Article** | Garbarski D | The measurement of gender expression in survey research | Gender expression, self-appraised and reflected gender expression | Gender expression: How individuals present their gender through appearance and behavior, either as self-perception or as perceived by others. Self-appraised gender expression: A person’s own assessment of their gendered appearance and behavior. Reflected gender expression: How a person believes others perceive their gendered appearance and behavior. | Primary approach (hypothesis-driven/literature-driven), examining survey measurement validity and reliability in assessing gender expression. |
| **Journal Article** | Hentschel T,Heilman ME,Peus CV | The multiple dimensions of gender stereotypes: A current look at men's and women's characterizations of others and themselves | Gender stereotypes, self-stereotyping, agency, communality | Gender stereotypes: Generalized beliefs about the characteristics of men and women. Self-stereotyping: The extent to which individuals apply gender stereotypes to themselves. Agency: Traits associated with assertiveness, competence, and independence. Communality: Traits associated with warmth, sociability, and emotional sensitivity. | Primary approach (hypothesis-driven/literature-driven), using established gender theories to measure multi-dimensional gender stereotypes. |
| **Journal Article** | Lombardi E,Banik S | The utility of the two-step gender measure within trans and Cis populations | Gender identity, sex assigned at birth, gender expression | Gender identity: A person’s self-identified gender, distinct from sex assigned at birth. Sex assigned at birth: The classification given to an individual at birth based on external anatomy. Gender expression: The way a person presents their gender, which may or may not align with their gender identity. | Primary approach (hypothesis-driven/literature-driven), validating the Two-Step Gender Measure for differentiating between trans and cis populations. |
| **Journal Article** | Kachel S,Steffens MC,Niedlich C | Traditional Masculinity and femininity: Validation of a new scale assessing gender roles | Gender roles, masculinity, femininity, gender-role self-concept | Gender roles: Socially constructed expectations regarding masculinity and femininity. Masculinity and femininity: Conceptualized as traditional constructs encompassing appearance, interests, behaviors, and self-identity. Gender-role self-concept: A higher-order construct reflecting how individuals perceive themselves regarding gender-typical characteristics. | Primary approach (hypothesis-driven/literature-driven), integrating gender role theory and stereotype research to construct a new measure. |
| **Journal Article** | Bauer GR,Braimoh J,Scheim AI,Dharma C | Transgender-inclusive measures of sex/gender for population surveys: Mixed-methods evaluation and recommendations | Gender identity, gender expression, sex assigned at birth, lived gender | Gender identity: Self-reported identity (e.g., male, female, non-binary, trans). Gender expression: How individuals present their gender in social interactions. Sex assigned at birth: The sex (male or female) recorded on an individual’s birth certificate. Lived gender: The gender in which an individual presents themselves in daily life. | Primary approach (hypothesis-driven/literature-driven), testing two existing sex/gender measures and proposing a new Multidimensional Sex/Gender Measure (MSGM) for population surveys. |
| **Journal Article** | Abdel-Sayyed A,Hoang KN,Turk T,Xu L,Fujiwara E | Validating the Stanford Gender-Related Variables for Health Research (SGVHR) in a Canadian population | Gender norms, gender-related traits, gender relations | Gender norms: Cultural expectations regarding behaviors appropriate for specific genders. Gender-related traits: Personality and behavioral characteristics that interact with gender norms, such as independence and risk-taking. Gender relations: The impact of gender on social interactions, discrimination, and caregiving responsibilities. | Tertiary approach (adaptation), validating the Stanford Gender-Related Variables for Health Research (SGVHR) in a Canadian population. |
| **Journal Article** | Komlenac N,Siller H,Bliem HR,Hochleitner M | Validation of the internal structure of a German-language version of the Gender Role Conflict Scale - Short Form | Masculine gender role conflict | Masculine Gender Role Conflict (GRC): Stress or discomfort experienced when behaviors conflict with masculine norms. Four patterns of GRC (O’Neil et al., 1986): Success, Power, and Competition (SPC): Constant obsession with outperforming others. Restrictive Emotionality (RE): Avoidance of emotional expression. Restrictive Affectionate Behavior Between Men (RABBM): Avoidance of expressing positive affection toward other men. Conflict Between Work and Family Relations (CBWFR): Prioritizing work over personal and family life. | Tertiary approach (adaptation), translating and validating the Gender Role Conflict Scale - Short Form (GRCS-SF) in German. |
| **Journal Article** | Tibubos AN,Otten D,Beutel ME,Brähler E | Validation of the personal attributes questionnaire-8: Gender expression and mental distress in the German population in 2006 and 2018 | Gender Expression (Masculinity and Femininity) | The study defines gender expression as traits reflecting masculinity and femininity, based on the Personal Attributes Questionnaire (PAQ), conceptualizing masculinity as agency/instrumentality and femininity as expressivity/communion. | Primary approach – The PAQ-8 was developed through scale reduction of the PAQ based on psychometric analyses from an existing dataset. |
| **Journal Article** | Levant RF,Alto KM,McKelvey DK,Richmond KA,McDermott RC | Variance composition, measurement invariance by gender, and construct validity of the Femininity Ideology Scale-Short Form | Traditional femininity ideology (TFI) | Traditional femininity ideology (TFI) is defined as an individual’s internalization of dominant cultural beliefs regarding appropriate roles for girls and women. It is theorized within the Gender Role Strain Paradigm (GRSP), which suggests that gender ideologies shape socialization and have implications for stress, strain, and conflict. | A primary (hypothesis-driven/literature-driven) approach. The original Femininity Ideology Scale (FIS) was constructed based on gender role strain theory, feminist theory, and prior literature, followed by factor analyses to refine the scale. |
